# Warning Signals of Post-Exertional Malaise in Myalgic Encephalomyelitis/Chronic Fatigue Syndrome: A Retrospective Analysis of 197 Patients

**DOI:** 10.3390/jcm10112517

**Published:** 2021-06-07

**Authors:** Alaa Ghali, Carole Lacout, Maria Ghali, Aline Gury, Estelle Delattre, Christian Lavigne, Geoffrey Urbanski

**Affiliations:** 1 Department of Internal Medicine and Clinical Immunology, Angers University Hospital, 49933 Angers, France; carole.lacout@chu-angers.fr (C.L.); aline.gury@chu-angers.fr (A.G.); estelle.delattre@chu-angers.fr (E.D.); chlavigne@chu-angers.fr (C.L.); geoffrey.urbanski@chu-angers.fr (G.U.); 2 Department of general medicine, Faculty of Medicine of Angers, 49045 Angers, France; maria.ghali@yahoo.fr

**Keywords:** myalgic encephalomyelitis/chronic fatigue syndrome, post-exertional malaise, warning signal, prevention

## Abstract

Post-exertional malaise (PEM), the key feature of myalgic encephalomyelitis/chronic fatigue syndrome (ME/CFS), is characterized by baseline symptom exacerbation after exposure to a stressor, and some patients can experience new or non-typical symptoms. We hypothesized that new or non-typical symptoms occurring long enough before onset of baseline symptom exacerbation could be warning signals predicting PEM. Adult ME/CFS patients who attended the internal medicine department of Angers University Hospital (France) between October 2011 and December 2019 were included in a retrospective medical records review. Patients who experienced one or more new or non-typical symptoms before baseline symptom exacerbation were compared with the rest of the study population for PEM features, epidemiological characteristics, fatigue features, and comorbidities. New or non-typical symptoms preceded baseline symptom exacerbation in 27/197 (13.7%) patients, and the most frequent ones were mood disorders (37%). When compared to the rest of the study population, only PEM intensity was significantly lower in these patients (*p* = 0.004), even after adjustment for sex and age at disease onset (*p* = 0.007). New or non-typical symptoms preceding baseline symptom exacerbation in some ME/CFS patients could be warning signals for PEM. Their identification could help preventing PEM occurrences or reducing their intensity leading to improving disease prognosis.

## 1. Introduction

Post-exertional malaise (PEM) is the key feature of myalgic encephalomyelitis/chronic fatigue syndrome (ME/CFS) and recent criteria require its presence to establish the diagnosis [1,2]. ME/CFS is a long-term and debilitating multisystem condition of unknown etiology affecting several millions of individuals worldwide [3]. It represents a significant public health issue due to the high levels of health care resource use by ME/CFS patients, and the loss of productivity in relation to the illness [4].

PEM is defined as an exacerbation of some or all baseline ME/CFS symptoms due to physical or cognitive stressors that were normally tolerated before disease onset. Worsening symptoms can include physical fatigue, cognitive difficulties, unrefreshing sleep, flulike symptoms, sore throat, pain, nausea, and other symptoms resulting in loss of stamina and/or functional capacity of ME/CFS patients [1,2,5]. Besides worsening of baseline symptoms, new or non-typical symptoms could emerge in some ME/CFS patients [2,5]. These symptoms differ from of those habitually experienced by patients after exposure to PEM stressors.

PEM onset is unpredictable since it may occur immediately after a stressor or be delayed by several hours or days. This onset delay helps distinguish ME/CFS from other diseases that manifest with severe fatigue and malaise without delayed onset, in particular multiple sclerosis [6] and systemic lupus erythematosus [7].

PEM has also an unexpected course due to the fact that severity and duration of symptoms are often out-of-proportion to the type, intensity, frequency, and/or duration of the stressor. Therefore, PEM severity varies greatly, not only between patients but also within the same patient [8,9]

PEM severely affects the quality of life of ME/CFS patients and was found to be significantly associated with disability [10], and predict a poorer outcome for patients [11]. Consequently, and because of the absence of treatment for ME/CFS, preventing PEM occurrence or at least reducing its severity constitutes the cornerstone of disease management.

The early symptoms and signs that precede the acute clinical phase of an illness are known under the term of “prodrome” [12]. The prodromal symptoms and signs can be non-specific, such as fever, malaise, headache, etc., as encountered in many infectious diseases, or specific to a particular disease such as migraine and psychosis. Nevertheless, they are not always present and vary between individuals, as in the case of migraine patients [13]. It was shown that defining prodromal symptoms and signs is useful in promoting early intervention and improving disease outcomes [14,15]. Taking the example of heart diseases, in which prodromal symptoms could be warning signals predicting an acute cardiac event such as acute myocardial infarction and sudden cardiac death [16,17], we hypothesized that new or non-typical symptoms emerging after exposure to one or more stressors sufficiently long enough before the onset of baseline symptom exacerbation could be warning signs of PEM occurrence in some patients. Thus, we aimed at identifying ME/CFS patients who experienced new or non-typical symptoms after a stressor, in order to describe those symptoms that precede the exacerbation of baseline symptoms.

## 2. Patients and Methods

### 2.1. Ethics

The study was approved by the ethics committee of Angers University Hospital (2018/46).

### 2.2. Study Population

We reviewed medical records of patients aged 18 years and above who attended the outpatient clinic of the internal medicine department of Angers University Hospital and were diagnosed as having ME/CFS on the basis of the International Consensus Criteria (ICC 2011) [1] between 31 October 2011 and 31 December 2019. 

### 2.3. Exclusion Criteria

Patients with an identifiable medical condition that could account for chronic fatigue were excluded, as well as those with primary psychiatric disorders or substance dependence. According to ICC 2011, the following comorbidities did not constitute an exclusionary condition: fibromyalgia, irritable bowel syndrome (IBS), Hashimoto’s thyroiditis, and reactive depression.

Patients’ medical records with missing data, especially about PEM assessment were also excluded.

### 2.4. Data Collection and Patients’ Grouping

All included patients were interviewed, physically examined and their histories taken. The medical history taken for each patient comprised a comprehensive analysis of PEM features, assessment of PEM severity, and evaluation of the fatigue level and its impact on patient activities. These data were carefully extracted from patients’ medical records together with epidemiological characteristics, baseline manifestations, and comorbidities.

We compared the proportion of patients who experienced the emergence of one or more new or non-typical symptoms after exposure to PEM stressors and before onset of baseline symptom exacerbation with the rest of the study population. 

### 2.5. Analysis of PEM Features

Each patient was questioned by the same physician about PEM features by means of a standardized questionnaire (Supplementary Figure S1) that was especially designed to collect data concerning PEM characteristics. These included the type of PEM stressors; the timing of onset of baseline symptom exacerbation, whether immediately after stressor or delayed; elapsed time between the exposure to a stressor and symptom exacerbation; PEM manifestations and worsened symptoms; potential emergence of new or non-typical symptoms that are different from habitual baseline symptoms and triggered by one or more PEM stressors; the time of occurrence of new or non-typical symptoms, whether they occur before onset of symptom exacerbation or not; the duration of PEM recovery. All patients were also asked to describe their feelings about their experience of PEM and responses were recorded in the patients’ own words.

### 2.6. Assessment of PEM Severity

PEM severity over the past month was assessed by means of the PEM item from the standardized self-reported questionnaire of Center for Disease Control and Prevention Symptom Inventory (CDC SI) [18]. Perceived frequency of PEM was rated on a 4-point scale (1 = a little of the time, 2 = some of the time, 3 = most of the time, 4 = all of the time), and its intensity was measured on a 3-point scale (1 = mild, 2 = moderate, 3 = severe). The intensity scores were converted into an equidistant score (0 = symptom not reported, 1 = mild, 2.5 = moderate, 4 = severe). The frequency and intensity scores were then multiplied to create the PEM severity score ranging from 0–16. 

### 2.7. Fatigue Assessment

Fatigue level was assessed in all patients by means of validated self-reported questionnaires; the fatigue scale (FS) [19] and the fatigue severity scale (FSS) [20]. The impact of fatigue on patient activities was assessed by the modified fatigue impact scale (MFIS) [21].

### 2.8. Statistical Analysis

Qualitative data were expressed as absolute number and percentage, and were compared with Chi-square test or Fisher’s test as appropriate for univariate analysis. Quantitative data were expressed as median and quartiles. The normality of the distribution for quantitative data was tested with the Shapiro–Wilk test, and the scedasticity by means of Levene’s test. Quantitative data were compared with Student’s t-test or Mann–Whitney test as appropriate for univariate analysis. Variables were then tested after adjustment for age at diagnosis and sex by means of binary logistic regression. The alpha risk was set at 5%. The analyses were performed using SPSS software v23.0 (IBM Corp, Chicago, IL, USA).

## 3. Results

**Figure 1:**
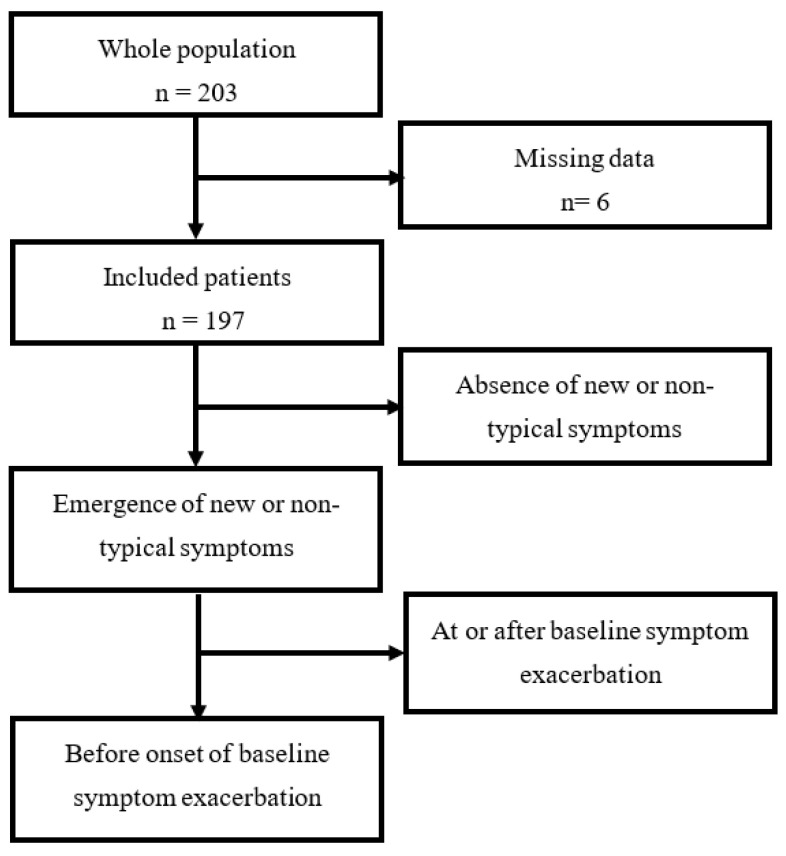
Flow chart.

The study population included 197 patients, 40 (20.3%) of whom experienced the emergence of one or more new or non-typical symptoms after exposure to PEM stressors. Among them, 27 (13.7%) experienced the occurrence of new or non-typical symptoms during the time elapsed between the exposure to a PEM stressor and the onset of baseline symptom exacerbation, i.e., PEM initiation (Figure 1). This prodromal phase lasted more than 3 hours in almost all patients (85.1%), and more than 24 hours in one patient.

The comparison between the proportion of patients who experienced new or non-typical symptoms after exposure to a PEM stressor and before onset of baseline symptom exacerbation and the rest of the study population showed that they were comparable for all variables except for PEM intensity, which was significantly lower in patients who experienced new or non-typical symptoms before the onset of their baseline symptom exacerbation (*p* = 0.004). This difference persisted after adjustment for sex and age at disease onset (*p* = 0.007). PEM frequency did not differ between both groups of patients (*p* = 0.93) (Table 1). Patients both diagnosed with comorbid reactional depression and/or fibromyalgia and exhibiting prodromal new or non-typical symptoms were comparable to the rest of the study population in terms of medication intake (Supplementary Table S1). 

In the group of patients who experienced new or non-typical symptoms before onset of baseline symptom exacerbation, the analysis of these symptoms showed that they were heterogeneous and varied between patients, however, they were almost constant for each patient. Table 2 shows the proportion of the different prodromal new or non-typical symptoms. 

Mood disorders, including emotional lability, irritable mood, lack of motivation, and depressed mood, were the most frequent new or non-typical prodromal symptom experienced by 10 (37%) patients before onset of baseline symptom exacerbation. Nausea was also frequent and preceded baseline exacerbation in 8 (29.6%) patients. In six (22.2%) patients, the prodromal phase of PEM was characterized by the sudden appearance of paresthesia that generally lasted from many hours to many days. For each patient, the topography of paresthesia was the same but differed largely between patients: hemi-facial, perioral, one-sided, or diffuse. Paresthesia was not part of baseline symptoms and no patients reported paresthesia in PEM-free periods. 

No association was found between the type of stressors and the nature of prodromal new or non-typical symptoms (Supplementary Table S2).

## 4. Discussion

PEM, the key feature of ME/CFS, is characterized by an exacerbation of one or more baseline ME/CFS symptoms after exposure to a stressor, and in some patients, the emergence of new or non-typical symptoms, which are not part of those habitually experienced by patients. We thus attempted to determine whether new or non-typical symptoms occurring before onset of baseline symptom exacerbation could be warning signals to predict PEM. The results of the current study showed that PEM intensity was significantly lower in patients who experienced new or non-typical symptoms before onset of baseline symptom exacerbation compared with the rest of the study population. This finding may suggest that new or non-typical symptoms occurring before onset of baseline symptom exacerbation in some ME/CFS patients could constitute an early warning signal for PEM onset.

One or more new or non-typical prodromal symptoms emerged in a proportion (13.7%) of ME/CFS patients some time before the onset of baseline symptom exacerbation and mostly regressed concomitantly with PEM recovery.

The comparison between this proportion of patients and the rest of the population showed that they were comparable in terms of epidemiological characteristics, fatigue levels, and comorbidities. Only PEM intensity was significantly lower in patients with prodromal new or non-typical symptoms even after adjustment for sex and disease onset. 

The frequency and intensity of PEM during the month preceding the evaluation were assessed in all patients by the standardized CDC SI self-reported questionnaire [18], which is a reliable and valid instrument for assessing symptoms associated with CFS, including PEM. It is one of two tools proposed by the National Academy of Medicine [2] for PEM assessment. The PEM severity score was obtained by multiplying the frequency score by the intensity score. Scoring the severity of PEM by considering both the frequency and the intensity of the PEM has the added merit of being able to ascertain what is more serious: a substantial PEM that appears irregularly or less significant PEM occurring repeatedly. 

In the current study, patients in whom new or non-typical symptoms emerged before the onset of baseline symptom exacerbation experienced PEM of lower intensity and similar frequency compared to the rest of the study population. The reduced PEM intensity could be explained by the fact that the appearance of new or non-typical symptoms sufficiently long enough before baseline symptom exacerbation in some patients had led them to modify or reduce activities to avoid more energy expenditure and further worsening of symptoms. Nevertheless, PEM frequency remained unchanged in these patients. Probably, there was not enough awareness of the relevance of warning signals among these patients in whom avoidance strategies were not sufficient enough and/or relatively delayed to avoid PEM occurrence. Therefore, PEM continued to occur with the same frequency. Raising awareness to warning signals may reduce both the frequency and intensity of PEM.

The analysis of a possible link between the type of stressors and the nature of prodromal new or non-typical symptoms found no association between a given stressor and the emergence of a particular new or non-typical symptom. However, with a small number of patients with prodromal new or non-typical symptoms, caution must be applied in interpreting this finding. To the best of our knowledge, this is the first study to identify symptoms that may predict PEM occurrence in ME/CFS patients.

Mood disorders were the most frequent prodromal new or non-typical symptoms. It is worth noting that none of the patients who experienced mood disorders had underlying psychiatric conditions, and that mood disorders regressed spontaneously throughout the PEM recovery period. One possible explanation for this finding might be that, in some ME/CFS patients, PEM and mood disorders share the same pathophysiological mechanisms. For instance, mitochondrial dysfunction including low ATP production, and increased oxidative stress and nitric oxide, was reported to be one of biological anomalies encountered in both PEM and mood disorders [22,23]. The exposure to a physical, cognitive, or emotional stressor could thus trigger both conditions, and mood disorders could precede baseline symptom exacerbation in some patients. Moreover, the concomitant improvement of both conditions could be evidence supporting this hypothesis.

Besides mood disorders, we observed other frequent prodromal new or non-typical symptoms, such as nausea, paresthesia, and headaches. Interestingly, despite their heterogeneity and large variability between patients, prodromal new or non-typical symptoms often remained the same for each patient, which reinforces their predictive power. 

One can wonder whether medication intake in patients with comorbid reactional depression or fibromyalgia to relieve symptoms can hinder the emergence of prodromal new or non-typical symptoms. In the current study, there were no significant differences in medication intake between patients both diagnosed with comorbid reactional depression and/or fibromyalgia and exhibiting prodromal new or non-typical symptoms when compared to the rest of the study population (Supplementary Table S1). Therefore, medication intake in these patients did not appear to affect the emergence of warning signals.

One question, however, remains unanswered: why a proportion of patients experienced new or non-typical symptoms before onset of baseline symptom exacerbation while others did not. Overall, the features and pathophysiology of PEM are still not well understood. Nevertheless, there is a growing body of evidence that PEM affects a variety of physiological systems. Studies that used maximal and submaximal exercise to elicit PEM showed altered cardiorespiratory responses [24], impaired pain regulation [25], impaired cognitive performance and affected brain function [26], altered gut microbiome and increased bacterial translocation [27], and immune involvement [28]. Mitochondrial dysfunction was also discussed as having a role in the nature of PEM [29], not only after exercise [30], but also in resting conditions [31]. Research addressing this issue must be conducted. 

### Limitations and Strengths

The subjectivity of PEM assessment could be a source of potential bias and it would be better to support this assessment with an objective evaluation. Currently, the only available way to objectively evaluate the PEM is the 2-day cardiopulmonary exercise test (CPET) that shows the loss of function and lack of recovery that occurs following exertion. Nevertheless, the disadvantage of CPET is that it may worsen the patient’s condition by triggering PEM. In addition, its systematic use in the research field is limited due to cost, expertise, and the level of severity for some participants [5]. Another source of weakness was the retrospective character of the study. 

On the other hand, we would like to highlight the sizeable number of the study population and the fact that all patients were examined, evaluated and diagnosed by the same physician, and underwent the same standardized procedure in terms of PEM and fatigue assessments. 

## 5. Conclusions

Taking warning symptoms into consideration could alert patients to the fact that they are going beyond their energy reserves and therefore have to reduce or stop their activity level in order to avert PEM occurrence. Consequently, it seems necessary to systematically look for prodromal new or non-typical symptoms, which are different from those usually experienced by ME/CFS patients. To do this, self-report questionnaires intended to assess PEM among these patients should include specific questions designed to examine the presence of the prodromal phase, explore warning symptoms that might occur after exposure to a PEM stressor and before onset of baseline symptom exacerbation, and specify whether or not these symptoms are different from those habitually experienced by patients. Similarly, and given that patients with ME/CFS usually show difficulties in predicting PEM occurrence, especially when the onset of baseline symptom exacerbation is delayed after the stressor, they must learn how to identify and recognize which warning signs, if any, may herald PEM onset, and be informed about how the impending threat of PEM could be avoided. To achieve this, it is important to facilitate access for patients to therapeutic educational programs, and raise awareness among primary care physicians about ME/CFS, in particular PEM issues.

## Figures and Tables

**Table 1:** Comparison between ME/CFS patients with prodromal new or non-typical symptoms ^a^ and the rest of the study population.

	Patients with Prodromal New or Non-Typical Symptoms ^a^	The Rest of the Study Population	*p*
Epidemiological characteristics			
Patients, n (%)	27 (13.7)	170 (86.3)	
Female, n (%)	21 (77.8)	125 (73.5)	0.64
BMI ^b^, kg/m^2^	22.2 (19.8–24.5)	23.1 (20–26.2)	0.40
Age at data collection, years	43 (33.5–50)	42 (34.3–50.5)	0.92
Age at onset, years	36 (24.5–43.5)	32 (25–40)	0.42
Age at diagnosis, years	40 (30.5–47.5)	40 (32.3–48)	0.81
Delay in diagnosis, months	43 (23–95)	47 (22–102)	0.40
Family history of fatigue	3 (11.1)	11 (6.5)	0.41
Fatigue assessment			
Fatigue severity scale	5.8 (4.5–6.5) (n = 24)	5.7 (5.1–6.4) (n = 128)	0.47
Fatigue scale	24 (19–26.8) (n = 18)	24 (20–28) (n = 138)	0.31
MFIS ^c^ physical	28.5 (26.3–31) (n = 18)	30 (26–33) (n = 137)	0.69
MFIS cognitive	30 (27.3–34.5) (n = 18)	26 (21–32) (n = 137)	0.07
MFIS psychosocial	6 (5–6) (n = 18)	6 (4.8–7) (n = 137)	0.88
PEM ^d^ assessment			
PEM frequency	3 (3–3)	3 (3–3)	0.93
PEM intensity	2.5 (2.5–4)	4 (2.5–4)	0.004
PEM severity	7.5 (7.5–12)	12 (7.5–12)	0.03
Comorbidities, n (%)	16 (59.3)	124 (72.9)	0.15
Reactional depression	7 (25.9)	64 (37.6)	0.24
Hashimoto’s thyroiditis	2 (7.4)	12 (6.9)	>0.99
Fibromyalgia	2 (7.4)	30 (17.6)	0.26
Irritable bowel syndrome	13 (48.1)	67 (39.4)	0.39

^a^ Symptoms occurring after exposure to a stressor and before onset of baseline symptom exacerbation; ^b^ Body mass index; ^c^ Modified fatigue impact scale; ^d^ Post-exertional malaise.

**Table 2:** Prodromal new or non-typical symptoms ^a^.

N° of Patients (%)	27/197 (13.7)
Headaches	6 (22.2)
Myalgia	2 (7.4)
Arthralgia	1 (3.7)
Paresthesia	6 (22.2)
Motor disturbances ^b^	2 (7.4)
Flulike	1 (3.7)
Nausea	8 (29.6)
Abdominal pain	1(0.5)
Palpitations	2 (7.4)
Collapse	3 (11.1)
Vertigo	3 (11.1)
Shortness of breath	3 (11.1)
Cold extremities	1 (3.7)
Mood disorders	10 (37)
Skin rash +/− pruritus	2 (7.4)

^a^ Symptoms occurring after exposure to a stressor and before onset of baseline symptom exacerbation; ^b^ Muscle weakness, twitching, poor coordination, unsteadiness.

## Data Availability

The data presented in this study are available on request from the corresponding author. The data are not publicly available due to privacy restrictions.

## Supplementary Materials

The following are available online at https://www.mdpi.com/article/10.3390/jcm10112517/s1, Figure S1: Assessment of Post-Exertion Malaise in Patients with Myalgic Encephalomyelitis/Chronic Fatigue Syndrome, Table S1: Medication intake in patients having myalgic encephalomyelitis/chronic fatigue syndrome with comorbid reactional depression and/or fibromyalgia, Table S2: Association between types of stressors and prodromal new or non-typical symptoms.
