# Supplementary material for: Warning Signals of Post-Exertional Malaise in Myalgic Encephalomyelitis/Chronic Fatigue Syndrome: A Retrospective Analysis of 197 Patients
Source: J Clin Med. 2021 Jun 7;10(11):2517. doi: 10.3390/jcm10112517 (PMC8201170; doi:10.3390/jcm10112517)
Supplement: Supplementary file 1 [file jcm-10-02517-s001.zip › jcm--supplementary-fig S1.pdf]

Figure S1: Assessment of Post-Exertion Malaise in patients with Myalgic Encephalomyelitis / Chronic Fatigue Syndrome

ANGERS UNIVERSITY HOSPITAL - INTERNAL MEDICINE DEPARTMENT
PATIENT: NAME ………………………. FIRST NAME ………………………. DATE OF BIRTH …. /…. /……..
ASSESSMENT DATE: …. /…. /……..

MPE ASSESSMENT

A. MPE FEATURES

A.1 MPE Stressors:

Q: What makes your symptoms worse?

Physical exertion: ☐
Cognitive exertion: ☐
Emotional stress: ☐
Orthostatism: ☐
Other stressors: ………………………………………………………………………………………
………………………………………………………………………………………

A.2 Timing of onset of symptom exacerbation after stressor:

Q: After a stressor, your symptoms get worse?

Immediately <1h: ☐
Delayed: ☐ 1-3h ☐ 3-6h ☐ 6-12h
☐ 12-24h ☐ >24h
Both: ☐

A.3 MPE manifestations:

A.3a Exacerbation of baseline ME/CFS symptoms:

Q: Please describe your symptoms that get worse after stressor

Physical fatigue: ☐
Cognitive difficulties: ☐
Brain fog: ☐
Unrefreshing sleep: ☐
Flu-like symptoms: ☐
Sore throat: ☐
Myalgia: ☐
Arthralgia: ☐
Nausea: ☐
Mood disorders ☐
Headache: ☐
Neurological: ☐
Other symptoms: ………………………………………………………………………………………

A.3b Emergence of new or non-typical symptoms (different from habitual baseline symptoms):

Q: Do you develop new symptoms different from that you usually experience?

☐ Yes ☐ No

Q: If yes, please describe these symptoms

....

....

Q: If yes, when do these new or non-typical symptoms appear?

☐ Before onset of baseline symptom exacerbation
☐ Concomitant with baseline symptom exacerbation
☐ After baseline symptom exacerbation

A.4 PEM duration (recovery time):

Q: How long does baseline symptom exacerbation last?

☐ 24h
☐ 1-3 days
☐ 3-7 days
☐ > 1week
☐ > 1month

A.5 MPE perception:

Q: How do you feel about your experience of PEM? (in the patient's own words)

....

<u>**B. MPE SEVERITY**</u> (CDC SI, Wagner et al. 2005)

B.1 MPE frequency:

Q: During the past month, how often have you had unusual fatigue after exertion?

☐ A little of the time
☐ Some of the time
☐ Most of the time
☐ All of the time

B.2 MPE intensity:

Q: During the past month, how bad was your unusual fatigue after exertion?

☐ Mild
☐ Moderate
☐ Severe

B.3 MPE Severity Score: ....
