# Supplementary material for: Warning Signals of Post-Exertional Malaise in Myalgic Encephalomyelitis/Chronic Fatigue Syndrome: A Retrospective Analysis of 197 Patients
Source: J Clin Med. 2021 Jun 7;10(11):2517. doi: 10.3390/jcm10112517 (PMC8201170; doi:10.3390/jcm10112517)
Supplement: Supplementary file 1 [file jcm-10-02517-s001.zip › jcm-1176016-supplementary.pdf]

Table S1. Medication intake in patients having myalgic encephalomyelitis/chronic fatigue syndrome with comorbid reactional depression and/or fibromyalgia

	**Patients with prodromal new or non-typical symptoms[a]**	**The rest of the study population**	**p**
Patients having reactional depression, n	7	64	
Medication: Antidepressants, n (%)	1 (14.3)	27 (42.2)	0.23
Anxiolytics, n (%)	3 (42.9)	19 (29.7)	0.67
Neuroleptics, n (%)	0 (0)	4 (6.3)	>0.99
Analgesics, n (%)	0 (0)	4 (6.3)	>0.99
Patients having fibromyalgia, n	2	30	
Medication: Antidepressants, n (%)	0 (0)	11 (36.7)	0.53
Anxiolytics, n (%)	0 (0)	10 (33.3)	>0.99
Neuroleptics, n (%)	0 (0)	0 (0)	>0.99
Analgesics, n (%)	0 (0)	7 (23.3)	>0.99

[a] Symptoms occurring after exposure to a stressor and before onset of baseline symptom exacerbation

Table 2. Association between types of stressors and prodromal new or non-typical symptoms

	**Prodromal new or non-typical symptoms**														
	**Headaches**	**Myalgia**	**Arthralgia**	**Paresthesia**	**Motor disturbances**	**Nausea**	**Palpitation**	**Collapse**	**Vertigo**	**Mood disorder**	**Flu-like symptoms**	**Abdominal pain**	**Cold extremities**	**Shortness of breath**	**Rash**
**Stressor type**															
Physical	> 0.99	> 0.99	> 0.99	> 0.99	> 0.99	0.30	> 0.99	> 0.99	> 0.99	> 0.99	> 0.99	> 0.99	> 0.99	> 0.99	> 0.99
Cognitive	> 0.99	> 0.99	0.33	> 0.99	> 0.99	0.38	> 0.99	0.25	> 0.99	0.09	> 0.99	> 0.99	0.33	0.25	0.54
Emotional	0.35	0.50	> 0.99	> 0.99	> 0.99	0.68	> 0.99	0.55	> 0.99	> 0.99	> 0.99	> 0.99	0.41	> 0.99	0.50
Infection	0.32	0.51	0.3	> 0.99	0.51	> 0.99	> 0.99	0.53	> 0.99	0.42	> 0.99	0.3	> 0.99	> 0.99	> 0.99
Orthostatism	0.59	0.4	> 0.99	0.59	0.4	> 0.99	0.4	0.55	> 0.99	> 0.99	> 0.99	> 0.99	0.22	0.55	> 0.99
Noise	> 0.99	> 0.99	> 0.99	0.4	> 0.99	0.51	> 0.99	> 0.99	> 0.99	0.52	> 0.99	> 0.99	> 0.99	> 0.99	> 0.99
Menses	0.06	> 0.99	> 0.99	> 0.99	0.34	0.62	> 0.99	> 0.99	> 0.99	0.33	> 0.99	0.19	> 0.99	0.47	> 0.99
Food	> 0.99	> 0.99	> 0.99	> 0.99	> 0.99	> 0.99	> 0.99	> 0.99	> 0.99	0.37	> 0.99	> 0.99	> 0.99	> 0.99	> 0.99
Chemicals	> 0.99	> 0.99	> 0.99	> 0.99	> 0.99	> 0.99	> 0.99	> 0.99	> 0.99	0.37	> 0.99	> 0.99	> 0.99	> 0.99	> 0.99
Humidity	0.22	> 0.99	> 0.99	> 0.99	> 0.99	> 0.99	> 0.99	> 0.99	> 0.99	> 0.99	> 0.99	> 0.99	> 0.99	0.11	> 0.99

The values in the table referred to the p-values according to Fisher's test
